# Genome-wide CRISPR Screening Reveals Pyrimidine Metabolic Reprogramming in 5-FU Chronochemotherapy of Colorectal Cancer

**DOI:** 10.3389/fonc.2022.949715

**Published:** 2022-07-12

**Authors:** Ya Niu, Xinyi Fan, Yaping Wang, Jiaxin Lin, Luchun Hua, Xiaobo Li, Ruizhe Qian, Chao Lu

**Affiliations:** ^1^ Department of Physiology and Pathophysiology of School of Basic Medical Sciences, Cancer Institute of Shanghai Cancer Center, Fudan University, Shanghai, China; ^2^ Department of Surgery, Huashan Hospital, Fudan University, Shanghai, China

**Keywords:** circadian rhythm, 5-fluorouracil, chronochemotherapy, CRISPR screening, BMAL1

## Abstract

**Objective:**

Disruption of the circadian rhythm is associated with cancer occurrence, response to chemotherapy, and poor prognosis. Thus, using internal clock-based chronotherapy to optimize the administration time may improve the therapeutic effects of anticancer drugs while reducing the side effects. Chronotherapy with 5-fluorouracil (5-FU) has been observed in colorectal cancer (CRC) for a long time, but its effect is under controversial and the mechanism remains unclear.

**Methods:**

Genome-wide clustered regularly interspaced short palindromic repeats (CRISPR) screening and RNA-sequencing were combined to identify the potential genes or pathways involved in 5-FU chronochemotherapy. Genetic deletion or overexpression of pyrimidine metabolic pathway genes were conducted to examine cellular viability with or without 5-FU *via* flow cytometry. Western blotting, qPCR, chromatin immunoprecipitation, gain-of-function and loss-of-function assays of several CRC cell lines *in vitro* and *in vivo* were used to elaborate and validate the mechanism of 5-FU chronotherapeutic effects.

**Results:**

Chronochemotherapeutic effects of 5-FU on CRC *in vivo* were verified. Furthermore, 5-FU chronochemotherapy related genes such as *UPP2*, *UCK2* and *UMPS* in the pyrimidine metabolic pathway were identified. Disturbance in these genes, especially *UMPS*, perturbs 5-FU treatment outcomes in CRC cells. Mechanistically, the core circadian gene, brain and muscle aryl hydrocarbon receptor nuclear translocator-like protein-1 (BMAL1), extensively regulate gene expression in pyrimidine metabolic pathway by binding to E-box element in the promoter region of key genes such as *UMPS* and perturb their enzymatic activities, thereby maintain diurnal efficacy of 5-FU in CRC cells.

**Conclusion:**

This study uncovered a new mechanism by which a core circadian gene BMAL1 increases the effectiveness of 5-FU by enhancing the expression and enzymatic activities of key genes in the pyrimidine metabolic pathway in CRC cells. The findings suggest a novel strategy for CRC chemotherapy by targeting chrono-modulated genes of the 5-FU metabolic pathway.

## Introduction

Circadian clocks are entrained by internal and external signals to regulate day-night fluctuations in an organism’s physiological and pathological events (1). The core clock machinery comprises a negative transcription-translation feedback loop. The core circadian proteins brain and muscle aryl hydrocarbon receptor nuclear translocator-like protein-1 (BMAL1) and circadian locomotor output cycles kaput (CLOCK) constitute a heterodimer that functions as a positive regulator, whereas cryptochrome and period heterodimerize to serve as negative regulators (2). The circadian clock is involved in regulating the hallmarks of cancer, including the cell cycle, DNA repair, and xenobiotic metabolism (3). Hence, drugs targeting these pathways have been explored for chronotherapy (4). Chronotherapy refers to the selection of an appropriate medication time based on the internal biological rhythm. This strategy is widely considered an effective way to maximize the benefits of the treatment while minimizing the toxicities (5). Nevertheless, the mechanism through which the clock regulates the effects of anticancer agents is unclear.

The drug 5-fluorouracil (5-FU) is a synthetic fluorinated pyrimidine analog that is widely used in the treatment of cancers including colorectal cancer (CRC), breast cancer, and respiratory and digestive tract cancers (6). This drug is mainly converted into active metabolites such as fluorodeoxyuridine monophosphate (FdUMP), which inhibits the action of thymidylate synthase (TYMS), a rate-limiting enzyme of DNA synthesis, ultimately leading to cell damage (7). However, acquired drug resistance is the main issue limiting its clinical application (8). Therefore, it is imperative to explore alternative strategies to improve 5-FU sensitivity.

Whether the chronotherapeutic effect of 5-FU is beneficial in CRC remains controversial. In subcutaneously transplanted sarcoma, *TYMS* is expressed in a circadian manner. Fourteen hours after light onset (HALO14), when TYMS activity is the lowest, 5-FU displays better therapeutic effects and fewer side effects than HALO22 (9). However, malignant processes could selectively alter the circadian rhythms that influence the chronotherapeutic effects in patients with cancer (10). Overall, a consistent statement on chronochemotherapy with 5-FU is absent in clinical research regarding CRC. For example, two multicenter randomized clinical trials have reported that a chronomodulated delivery of combination chemotherapy with oxaliplatin, 5-FU, and leucovorin is more effective and less toxic than constant administration in the treatment of metastatic CRC (11, 12). However, a larger study in Europe found “no beneficial effect of chronotherapy in the study population as a whole”, but it was simultaneously observed that male patients with CRC benefited from a chronomodulated scheme, indicating that chronotherapy may be sex-dependent and complicated (13). Therefore, there is an urgent need to understand the underlying mechanism explaining the intricate role of the biological clock in chronotherapy involving 5-FU.

In this study, we analyzed and compared the effects of administering 5-FU at HALO16 and HALO4 on the volume and weight of tumors in subcutaneously transplanted nude mice. In addition, we performed genome-wide CRISPR-based screening combined with RNA sequencing to identify the pathways involved in 5-FU chronochemotherapy. We further investigated the role of the core circadian gene *BMAL1* in regulating pyrimidine metabolic pathway-related genes, along with the underlying mechanism, and tested the effects of altering the expression of these genes on the sensitivity of CRC cells to 5-FU. Overall, our research revealed a new mechanism by which the circadian time system affects 5-FU chronochemotherapeutic efficacy in CRC, which may help increase drug efficacy.

## Materials and methods

### Cell Lines and Cell Culture

SW480 and SW620 human CRC cell lines were purchased from the Cell Bank of the Chinese Academy of Sciences (Shanghai, China). The HCT116 human CRC cell lines were a gift from Southern Medical University (Guangzhou, China). Cells were maintained as previously described (14).

### Circadian Rhythm Induction

Cells were seeded in dishes or well plates, the medium was removed from confluent cultures of CRC cells after starvation for 24 h, and cells were treated with 50% horse serum (Gibco, MA, USA) for 3 h followed by replacement with fresh medium (the time of addition of 50% horse serum was ZT0) (15). The cells did not receive any further medium changes from this point onward until the time of harvest. Individual plates were harvested for total RNA and protein extraction. Synchronized cells in 6-well or 96-well plates with or without 5-FU were used for flow cytometry analysis.

### Xenografted Tumor Model

All male BALB/c nude mice (4 weeks old) were purchased from GemPharmatech (Nanjing, China). All mice were kept under 12:12 LD conditions with the light on from 8 a.m. to 8 p.m. and fed with water and antibiotic-free food *ad libitum*. All animal experiments were performed with the approval of the Institutional Animal Care and Use Committee. HCT116 cell suspensions [5 × 106 cells mixed in 200 μL containing serum-free medium and growth reducing Matrigel (1:1)] were subcutaneously injected into the backs of mice. One week after cell inoculation, mice were treated with 5-FU (30 mg kg^−1^, twice a week) or PBS at the indicated time points for 4 weeks. Tumor weight and volume were monitored once a week. At the endpoint of the experiment, the mice were sacrificed to compare tumor weight and volume.

### CRISPR-Cas9 Library Screening and Cell Transduction

The SW480 cell line was transduced at a multiplicity of infection of ~ 0.5 with lentivirus containing a genome-wide lentiviral human CRISPR knockout guide library with 58 028 single-guide RNAs (sgRNAs) from Genscript (Nanjing, China) with approximately 300-fold representation (cells per construct). Puromycin (1 μg/mL) was added to the cells after transduction for 24 h and maintained for 7 days. Subsequently, 2 × 10^7^ cells were collected for baseline genomic DNA analysis. The remaining cells were split into four dishes, synchronized using horse serum, and treated with 5-FU (500 μM) or DMSO at the indicated time for 36 h. After drug treatment, 2 × 10^7^ cells were collected from either the drug group or the DMSO control group for analysis.

### Data Processing and Initial Analysis

MAGeCK software (v0.5.1) was used for data analysis (16). Sample reads were mapped to the reference sgRNA library with mismatch option as 0 using MAGeCK. Median normalization was performed to adjust for library sizes and read counts. Raw reads and normalized reads were obtained as described by Zhang *et al.* (17). With the standard reads acquired above, the data were analyzed as described.

### Data Analysis

In brief, considering the variation in transfection and knockdown efficacy, we focused on sgRNAs rather than genes. Then, data obtained from ZT16 DMSO, ZT16 5-FU, ZT28 DMSO, and ZT28 5-FU groups were labeled datasets A, B, C, and D, respectively. First, the nontargeting sgRNAs and sgRNAs with reads of 0 reads were deleted before adding 1 read per sgRNA in each group. Therefore, the A1, B1, C1, and D1 datasets were obtained. Second, sgRNAs were selected with reads that showed changes < 0.5 or ≥ 2 times when compared the ZT16 5-FU group with the ZT16 DMSO group, and then, the datasets were named A2 and B2 respectively. Third, genes showing relatively high confidence that corresponded to the same sgRNA in B2 (named B2’ dataset) and the significant genes corresponding to the sgRNA that showed a ratio of reads B2/A2 ≥ 5 times (named B2’’ dataset) were selected. B2’ was combined with B2’’ to obtain the B3 dataset. Similarly, the dataset including genes corresponding to the same sgRNAs in the A2 dataset was named A2’. The A2’’ dataset included the significant genes corresponding to the sgRNAs showing a read ratio of B2/A2 < 0.2. The A3 dataset was obtained by combining A2’ with A2’’ (A3 = A2’ + A2’’). The D3 and C3 datasets were obtained in the same way. Finally, B3 was intersected with D3, and the datasets containing the unique data points from B3 and D3 were labeled as B4 and D4, respectively. Likewise, the A4 and C4 datasets were obtained.

### Statistical Analysis

Data were assessed using a T test or one-way ANOVA. The results are summarized using the mean ± standard deviation (SD). P-value < 0.05 was identified as statistically significant and all statistical data were analyzed with GraphPad Prism 8.0 software.

## Results

### The Therapeutic Effects of 5-FU in CRC Are Rhythmic *In Vivo*

**Figure 1:**
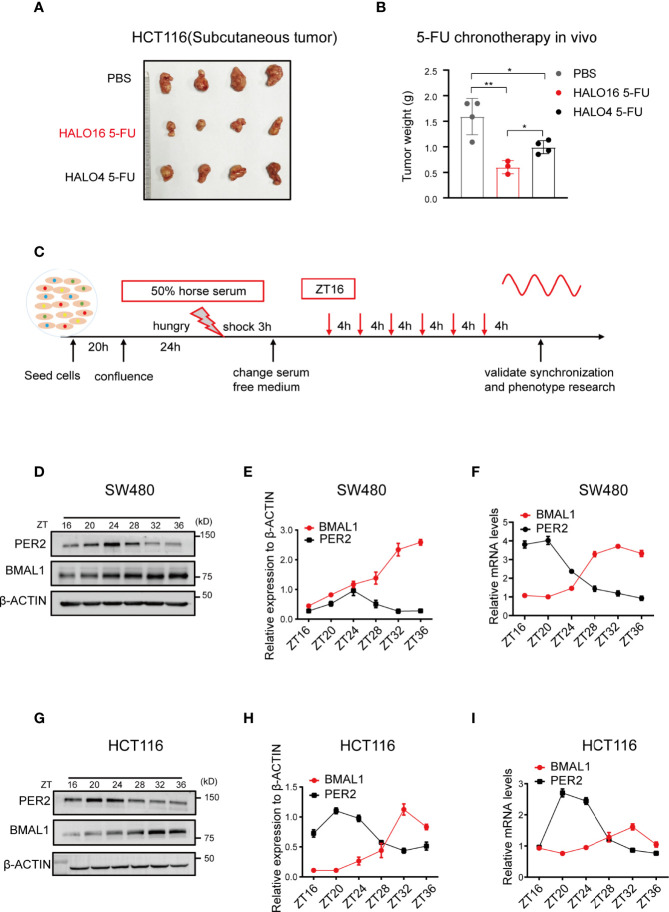
The therapeutic efficacy of 5-FU in CRC are rhythmic *in vivo.* ***(*A, B*)*** HCT116 cells were subcutaneously injected into mice to observe 5-FU chronochemotherapy in cohort 1. One week after cell inoculation, the mice were treated with 5-FU (30 mg kg^-1^, twice a week) or PBS at the indicated time points for four weeks. (n = 4 unpaired t test). **(C)** Schematic diagram for resetting the circadian oscillations in cultured cells by horse serum. Cells were collected every 4 h for a total of 24 h at the indicated time points. ZT, zeitgeber time (literally, time given or time cue) refers to environmental variables that act as circadian time cues. **(D–F)** The expression of BMAL1 and PER2 at the indicated time points in SW480 cells after synchronization using Western blotting and qPCR (n = 3). **(G–I)** The expression of BMAL1 and PER2 at the indicated time points in HCT116 cells after synchronization using Western blotting and qPCR (n = 3).

Considering the lack of a consistent statement, we investigated the pharmacological variation in 5-FU chronochemotherapy in CRC using *in vivo* assays. Nude mice with subcutaneous tumors were injected intraperitoneally twice a week with 30 mg kg^−1^ 5-FU at HALO4 and HALO16 for a total of 4 weeks after synchronization under 12 h light and 12 h dark (12:12 LD) conditions for two weeks. The results of the 5-FU treatment in the two cohorts showed that the volume and weight of xenografts in the HALO16 group were significantly smaller than those in the HALO4 group (**Figures 1A, B**; **Supplementary Figures 1A–C**), along with lower intertumor volume variability. These data showed that 5-FU had a chronochemotherapeutic effect on CRC.

### Genome-Wide CRISPR Screening Identifies the Pyrimidine Metabolic Pathway Involved in 5-FU Chronomodulated Efficacy in CRC

**Figure 2:**
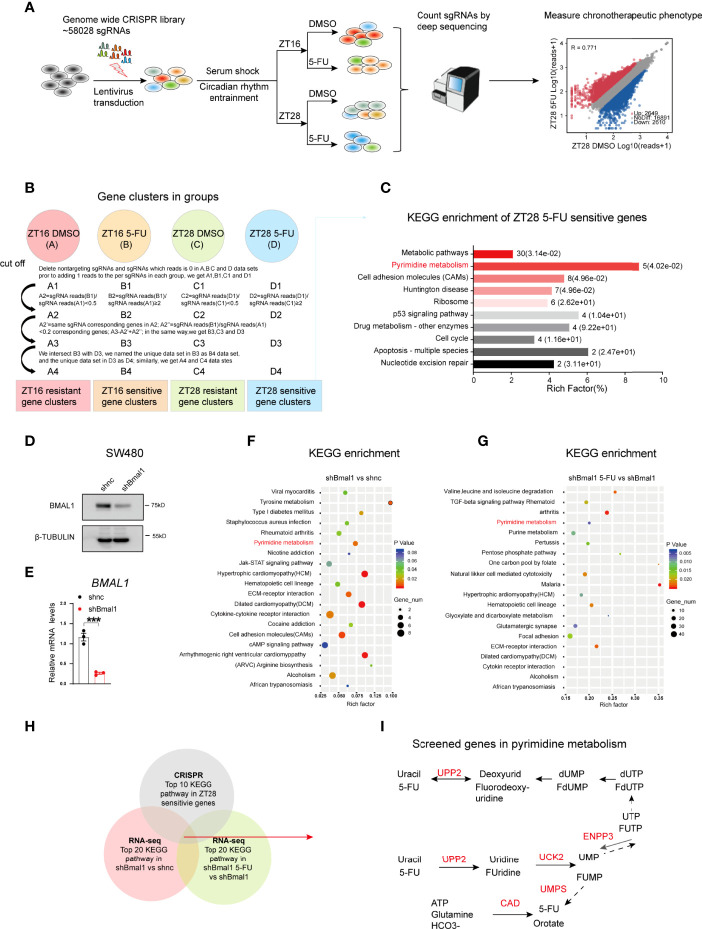
Genome-wide CRISPR screening reveals the pyrimidine metabolic pathway involved in 5-FU chronomodulated efficacy in CRC. **(A)** Diagram depicting the genome-wide CRISPR screening process. **(B)** Analytical process of the ZT16 DMSO, ZT16 5-FU, ZT28 DMSO, and ZT28 5-FU groups. The ZT16 5-FU group indicates 5-FU treatment at ZT16, and the ZT16 DMSO group indicates DMSO treatment at ZT16. The same was true for the ZT28 5-FU and ZT28 DMSO groups. **(C)** KEGG pathway analysis of ZT28-sensitive genes. The numbers on the right of the bars indicate the ratio of enriched hits, and the content in the brackets indicates the corresponding p value. **(D, E)** The protein expression levels of BMAL1 after knocking down BMAL1 in SW480 cells *via* western blotting and qPCR. (n = 3, unpaired t test). *** indicates p < 0.005. **(F)** KEGG pathway analysis of significant genes in the shBmal1 versus shnc groups. **(G)** KEGG pathway analysis of significant genes in the shBmal1 5-FU versus shBmal1 groups. **(H)** Venn diagram showing intersecting pathways in genome-wide CRISPR and RNA-seq screening. **(I)** Schematic diagram showing the location of screened genes in the pyrimidine metabolic pathway. They are *UPP2*, *UCK2*, *ENPP3*, *UMPS*, carbamoyl-phosphate synthetase 2, aspartate transcarbamoylase, and dihydroorotase (*CAD*). Briefly, 5-FU is converted to fluorouridine monophosphate (FUMP) either directly through orotate phosphoribosyl transferase (UMPS) or indirectly through the sequential action of UPP2 and UCK. ENPP3 is a phosphohydrolase that hydrolyzes nucleotide triphosphates into monophosphate nucleotides.

To investigate the mechanism underlying the chronochemotherapeutic effects of 5-FU in CRC, we performed genome-scale CRISPR screening using the human GeCKO library B in SW480 cells with or without 5-FU. This library contained 58 028 sgRNAs, including three sgRNAs per gene and 1 000 nontargeting controls (18). We successfully synchronized the biological rhythms of various CRC cell lines, such as SW480, HCT116, and SW620, using horse serum (**Figures 1C–I**; **Supplementary Figures 1D–F**). We found that *TYMS* expression was rhythmic in synchronized CRC cells (**Supplementary Figure 2**). Hence, 5-FU was administered at zeitgeber 16 (ZT16, when TYMS expression was near the peak) and ZT28 (when TYMS expression was near the trough). Next, we sequenced the genomic DNA from each population and analyzed the sgRNA distribution between groups using MAGeCK (**Figure 2A**). The sgRNA mapping rate was > 75% in the five groups (**Supplementary Figure 3A**) with considerable uniformity and correlations among the sequencing samples (**Supplementary Figures 3B, C**) and a coverage of approximately 100–300 × in most sgRNA sequences (**Supplementary Figure 3D**). Data were first analyzed using MAGeCK (**Supplementary Figures 4A–C**). However, due to complicated circadian factors and drug factors (19), the data were analyzed using a method that better fit our model (**Figure 2B**). Kyoto Encyclopedia of Genes and Genomes (KEGG) analysis showed that ZT28 5-FU sensitivity genes were enriched in cancer-related hallmark pathways. These pathways included metabolic pathways, pyrimidine metabolism, P53 signaling pathways, cell cycle and nucleotide excision repair (**Figure 2C**). Among these, pyrimidine metabolism was the most significantly enriched pathway.

To selectively identify the genes or pathways that are possibly associated with chrono-dependent 5-FU efficacy, we performed RNA sequencing of shBmal1 and shnc SW480 cells in the presence or absence of 5-FU (**Figures 2D–G**). Surprisingly, the pyrimidine metabolic pathway was also involved in the regulation of the BMAL1-mediated 5-FU response. Specifically, genes encoding five enzymes of the pyrimidine metabolic pathway were identified: uridine phosphorylase 2 (*UPP2*), uridine-cytidine kinase 2 (*UCK2*), ectonucleotide pyrophosphatase/phosphodiesterase 3 (*ENPP3*), uridine monophosphate synthetase (*UMPS*), and carbamoyl-phosphate synthetase 2, aspartate transcarbamoylase, and dihydroorotase (**Figures 2H, I**). Taken together, these results highlight the pivotal role of the pyrimidine metabolic pathway in 5-FU chronochemotherapy.

### Genetic Deletion of Pyrimidine Pathway-Related Genes Promotes Robust 5-FU Resistance in CRC Cells

**Figure 3:**
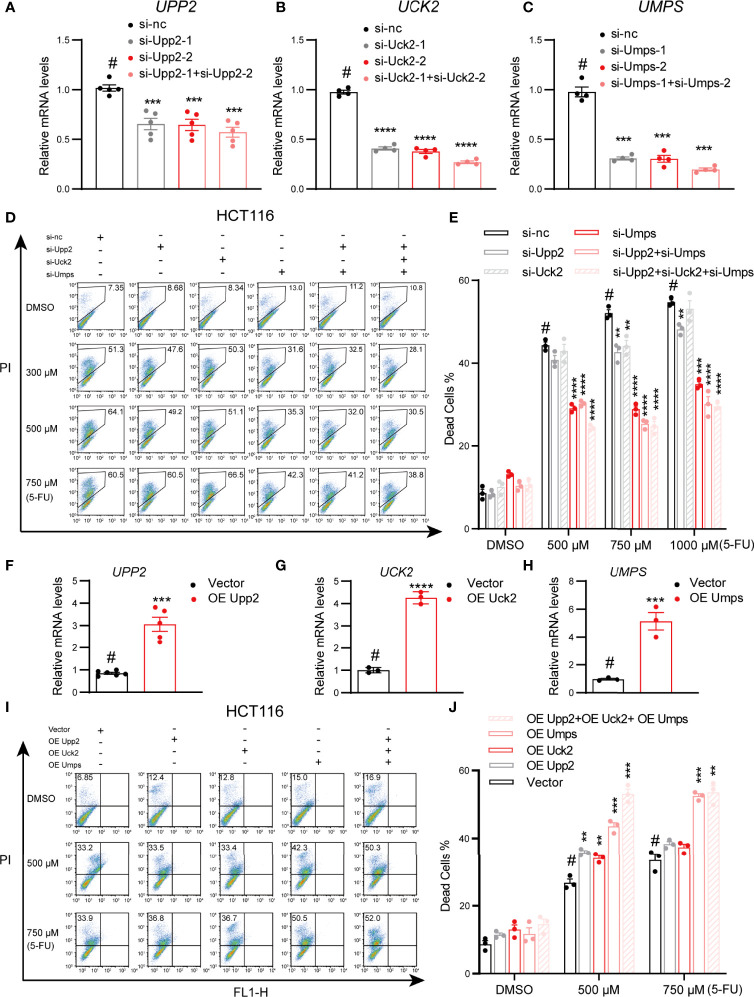
Genetic deletion of pyrimidine pathway genes promotes robust 5-FU resistance in HCT116 cells. **(A–C)** Validation of the knockdown efficiency of *UPP2* by qPCR in HEK293T, and validation of the knockdown efficiency of *UCK2* and *UMPS* in by qPCR in HCT116 cells. **(D, E)** Dead cells were determined by flow cytometry of HCT116 cells knocking down pyrimidine metabolic pathway genes after treatment with 5-FU (0, 300 μM, 500 μM, 750 μM, 48 h). (n = 3, one-way ANOVA per concentration). **(F–H)** Validation of the overexpression efficiency of *UPP2*, *UCK2* and *UMPS* by qPCR in HCT116. **(I, J)** Dead cells were determined by flow cytometry of HCT116 cells overexpressing pyrimidine metabolic pathway genes after treatment with 5-FU (0, 500 μM,750 μM,48 h). (n = 3, one-way ANOVA per concentration). ** indicates p < 0.01, *** indicates p < 0.005, **** indicates p< 0.001 versus si-nc (#) or vector (#), ns indicates there is no significant.

To determine whether pyrimidine pathway-related genes affect 5-FU sensitivity in CRC cells, we knocked down and overexpressed *UPP2*, *UCK2* or *UMPS* in SW480 and HCT116 cell lines before administration of 5-FU. Then, cell viability was measured *via* flow cytometry. The results showed that the downregulated expression of any of these three genes conferred partial resistance to 5-FU, most prominently in the case of *UMPS*. Moreover, simultaneous repression of two or three of these genes conferred higher resistance than single-gene knockdown. In addition, the resistance effects were dose-dependent (**Figures 3A–E**). Accordingly, overexpression of pyrimidine metabolic genes conferred sensitivity to 5-FU (**Figures 3F–J**). Moreover, a consistent phenotype was found in SW480 cells (**Supplementary Figure 5**). These results indicated the importance of pyrimidine metabolic genes in 5-FU treatment efficiency.

### BMAL1 Extensively Regulates Gene Expression in the Pyrimidine Metabolic Pathway and Stimulates Pyrimidine Metabolism in CRC Cells

Analysis of CircaDB (20) revealed that the genes identified in our study, as well as certain other genes involved in 5-FU metabolism mediated by the pyrimidine metabolic pathway were expressed in a circadian rhythm (**Supplementary Figure 6**). These data indicated that the genes involved in pyrimidine metabolism were clock-controlled.

**Figure 4:**
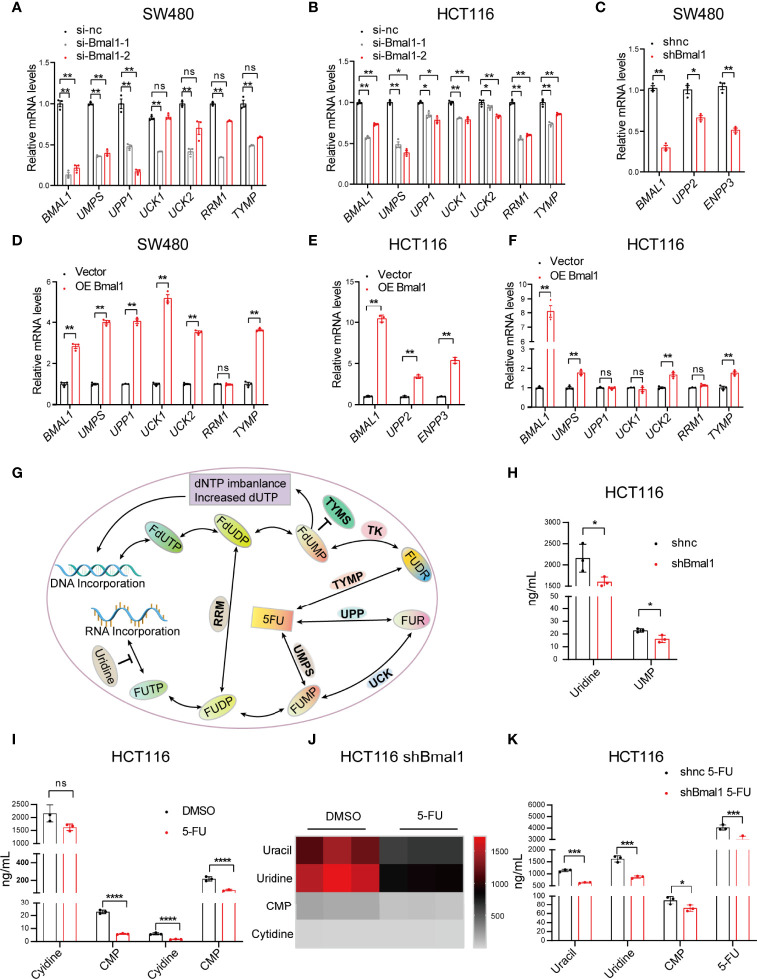
BMAL1 extensively regulates gene expression in the pyrimidine metabolic pathway and stimulates pyrimidine metabolism in CRC cells. **(A–C)** Confirmation of the changes in pyrimidine pathway genes related to 5-FU metabolism, while BMAL1 was deregulated in SW480 and HCT116 cells by qPCR. **(D–F)** Confirmation of the changes in pyrimidine pathway genes related to 5-FU metabolism, while BMAL1 expression was upregulated in SW480 and HCT116 cells, as shown by qPCR. * indicates p < 0.05, ** indicates p < 0.01 versus si-nc or vector, ns indicates there is no significance. **(G)** Schematic depicting the mechanism of 5-FU. In brief, 5-FU is converted into various fluoronucleotide derivatives that disrupt RNA and DNA syntheses by incorporation into RNA and DNA, thus exerting its cytotoxic effects in cells. The mechanism of 5-FU activation is conversion to fluorouridine monophosphate (FUMP), either directly through orotate phosphoribosyltransferase (UMPS) or indirectly by fluorouridine (FUR) through the sequential action of uridine phosphorylase (UPP) and uridine kinase (UK). Other enzymes depicted are thymidine phosphorylase (TYMP), thymidine kinase (TK), thymidylate synthase (TYMS) and ribonucleotide reductase (RRM). Other metabolites indicated are fluorodeoxyuridine (FUDR), fluorodeoxyuridine monophosphate (FdUMP), fluorouridine diphosphate (FUDP), fluorodeoxyuridine diphosphate (FdUDP), fluorouridine triphosphate (FUTP), fluorodeoxyuridine triphosphate (FdUTP), deoxynucleotide (dNTP), and deoxyuridine triphosphate (dUTP). **(H)** The content of pyrimidine metabolites in shBmal1 and shnc cells measured by LC-MS. **(I)** The content of pyrimidine metabolites in shnc cells after 5-FU treatment (500 μM). **(J)** Heatmap showing the contents of uracil, uridine, cytidine monophosphate (CMP), and cytidine in shBmal1 cells after 5-FU treatment (500 μM). **(K)** Detection of the contents of uracil, uridine, CMP, and cytidine in shBmal1 cells after 5-FU treatment using LC-MS (n = 3, unpaired t test). * indicates p < 0.05, *** indicates p < 0.005, **** indicates p < 0.001.

To further validate whether the pyrimidine metabolic pathway is regulated by the circadian clock, we examined the changes in the expression of pyrimidine metabolic pathway genes, e.g., *UMPS*, uridine phosphorylase 1 (*UPP1*), *UPP2*, uridine-cytidine kinase 1 (*UCK1*), *UCK2*, ribonucleotide reductase (*RRM1*), thymidine phosphorylase (*TYMP)*, and *ENPP3* after overexpression or knockdown of *BMAL1* in SW480 and HCT116 cells. We found that the mRNA expression levels of these genes decreased significantly after *BMAL1* knockdown, and the decrease was most prominent for *UMP*S (**Figures 4A–C**). Moreover, the mRNA expression levels of these genes increased significantly after *BMAL*1 overexpression (**Figures 4D–F**). These results indicated that these genes were affected by the core circadian clock gene *BMAL1*.

To elucidate whether BMAL1 regulates the activities of pyrimidine metabolic enzymes, we monitored the pyrimidine metabolite content in the shBmal1 and shnc cells with or without 5-FU using liquid chromatography coupled with mass spectrometry (LC-MS) (**Figure 4G**). The shBmal1 cells showed lower concentrations of uridine monophosphate (UMP), uracil, uridine, cytidine monophosphate (CMP), and cytidine than the shnc cells. Moreover, the reduction in the levels of the pyrimidine metabolites UMP and uridine was statistically significant (P < 0.05) (**Figure 4H**), suggesting that BMAL1 was involved in their regulation. Since 5-FU is a pyrimidine analog, its pharmacological activities are anticipated to be similarly affected by BMAL1. As predicted, 5-FU independently modulated the levels of pyrimidine metabolites, and the disturbance was more prominent when BMAL1 was knocked down (**Figures 4I, J**). Compared with those in the 5-FU-treated shnc cells, the uracil, uridine, CMP, and 5-FU levels in the shBmal1 5-FU cells were significantly lower (P < 0.05) (**Figure 4K**). These results suggested that BMAL1 affected pyrimidine metabolism and might influence chronochemotherapeutic function.

### BMAL1 Regulates *UMPS* Expression by Binding to the E-box at the *UMPS* Promoter

**Figure 5:**
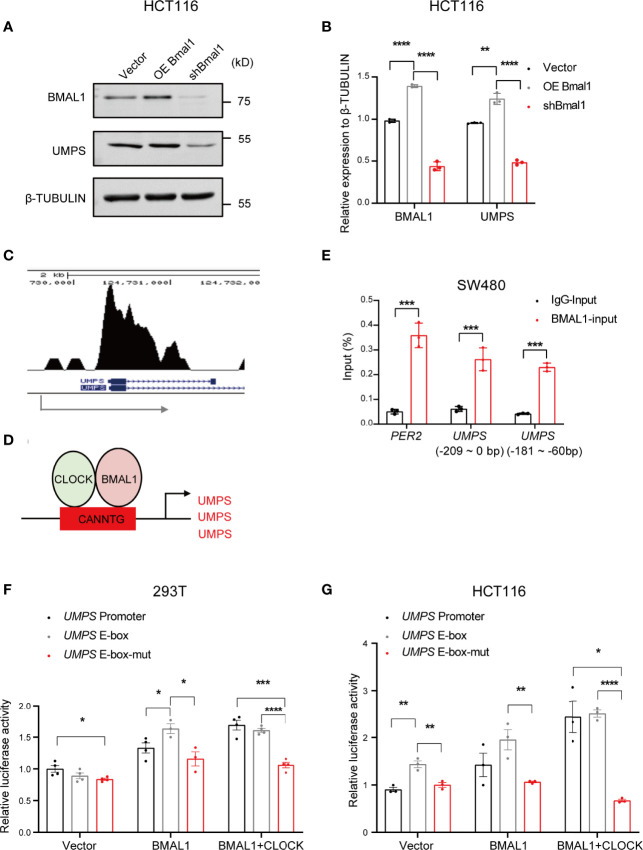
BMAL1 regulates *UMPS* expression by binding to the E-box in the *UMPS* promoter. **(A, B)** The protein expression levels of BMAL1 and UMPS upon knockdown or overexpression of BMAL1 in HCT116 cells *via* western blotting. **(C)** ChIP-seq of BMAL1 showed binding signals on the promoter region of *UMPS* in CRC LOVO cells. The blue arrow indicates the transcription start site (TSS) of the *UMPS* gene. **(D)** The schematic shows the CLOCK-BMAL1 binding sites at the *UMPS* promoter. **(E)** ChIP analysis of BMAL1 binding to the E-box region of *UMPS* in HCT116 cells. -186 bp represents 186 base pairs from the transcriptional start site of *UMPS*. **(F, G)** Luciferase reporter assays revealed transcriptional activation of UMPS in HEK 293 and HCT116 cells by BMAL1 or BMAL1+CLOCK. When the binding site was mutated, the activation was significantly decreased. * indicates p < 0.05, ** indicates p < 0.01, *** indicates p < 0.005, **** indicates p<0.001.

We further investigated how the circadian clock regulates pyrimidine metabolic genes and mediates the pharmacological activity of 5-FU. Our data conformed that UMPS expression in CRC cells was increased after overexpressing BMAL1 and were decreased after knocking down BMAL1 using western blotting (**Figures 5A, B**), suggesting that UMPS transcription and translation were controlled by BMAL1.

According to the genome-wide transcript profile, BMAL1 targets over 150 sites in the human genome, including all known clock genes and several genes regulating metabolism (21). BMAL1 is a core circadian transcription factor that regulates downstream genes by binding to E-box elements in their promoters (22). Since UMPS is a critical enzyme in the pyrimidine metabolic pathway, we hypothesized that BMAL1 activates the transcription of UMPS in CRC cells by binding to E-box elements within its promoter region to regulate pyrimidine metabolism. Indeed, ChIP-seq analysis showed that BMAL1 could bind to the promoter region of *UMPS* in CRC cells (**Figure 5C**). Moreover, ChIP-qPCR assays confirmed the presence of a BMAL1 binding site from -209 to 0 bp at the *UMPS* promoter from the transcriptional start site (**Figures 5D, E**). We then evaluated the transcriptional activation of *UMPS* by BMAL1 using luciferase assays. The wild-type *UMPS* promoter resulted in the highest transcriptional activity of the luciferase gene in the presence of BMAL1 or BMAL1+CLOCK. However, the *UMPS* promoter with a mutated E-box was unable to sufficiently trigger the transcription of the reporter gene with exogenous overexpression of BMAL1 with or without CLOCK (**Figures 5F, G**). These results indicate that BMAL1 regulates *UMPS* transcription by binding to the E-box within its promoter region.

### UMPS Enhances 5-FU Sensitivity Through BMAL1 in CRC Cells *In Vitro* and *In Vivo*

**Figure 6:**
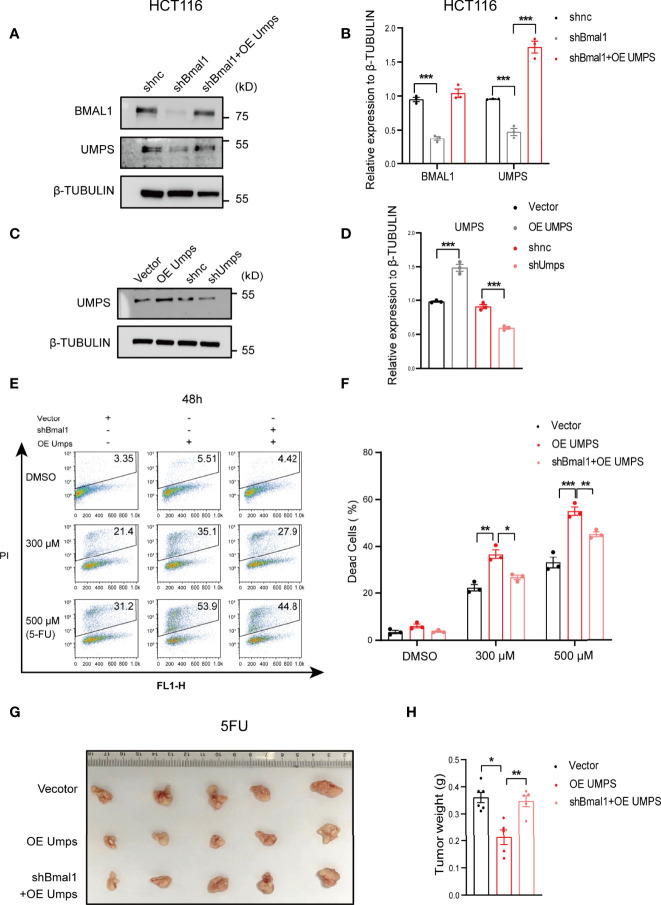
UMPS enhances 5-FU sensitivity through BMAL1 *in vitro* and *in vivo* in CRC cells. **(A, B)** The protein expression levels of BMAL1 and UMPS upon simultaneous knockdown of BMAL1 with or without overexpression of UMPS in HCT116 cells *via* western blotting. **(C, D)** The protein expression levels of UMPS upon knockdown or overexpression in HCT116 cells *via* western blotting. **(E, F)** Dead cells were determined by flow cytometry of HCT116 cells with simultaneous knockdown of BMAL1 with or without overexpression of UMPS after treatment with 5-FU (0, 300 μM, 500 μM, 750 μM, 48 h). (n = 3, one-way ANOVA per concentration). **(G, H)** OE Umps or shBmal1+OEUmps stable HCT116 cells were subcutaneously injected into mice. One week after cell inoculation, mice were treated with 5-FU (30 mg kg^−1^, twice a week) at HALO16 for 4 weeks. (n = 5 or 6, unpaired t test). After 4 weeks of treatment, the tumors were resected and analyzed. * indicates p < 0.05, ** indicates p < 0.01, *** indicates p < 0.005.

We then performed gain-of-function and loss-of-function analyses to determine whether the increase in 5-FU sensitivity caused by UMPS is mediated *via* BMAL1. 5-FU sensitivity declined in the shBmal1 group compared with that in the control group, whereas it increased in the shBmal1+OEUmps group, and the effects were dose-dependent (**Figures 6A–F**). In addition, we observed a decrease in the tumor weight of subcutaneous xenografts generated from the 5-FU-treated shBmal1+OEUmps CRC cells compared with that of the OE Umps group (**Figures 6G, H**). These observations suggest that the inactivation of UMPS caused by BMAL1 knockdown played a vital role in 5-FU resistance. Moreover, we noted that 5-FU sensitivity in the shUmps group was lower than that in the OEBmal1+shUmps group. Additionally, these effects were dose-dependent (**Supplementary Figure 7**). Taken together, these data indicated that UMPS, a key enzyme in the pyrimidine metabolic pathway, improved 5-FU sensitivity *in vitro* and *in vivo*, and that this phenomenon was regulated by BMAL1.

## Discussion

Many studies have focused on improving chronotherapy, but little provides a better understanding of the mechanism of the circadian clock in cancers. In this study, we used a genome-wide CRISPR-based screening approach to investigate the involvement of the pyrimidine metabolic pathway in 5-FU chronochemotherapy in CRC. Deletion of pyrimidine pathway-related genes confers 5-FU resistance in CRC cells. By analyzing CircaDB, we found that the expression of most pyrimidine metabolic pathway-related genes was periodic. Moreover, pyrimidine metabolic pathway genes such as *UPP2*, *UCK2* and *UMPS* were conformed to be regulated by BMAL1, among which *UMPS* was predominantly regulated by this mechanism. Mechanistically, BMAL1 binds to the E-box region of promoters to regulate their expression, which not only promotes 5-FU metabolism but also increases the 5-FU content in CRC cells. This phenomenon affects 5-FU pharmacological activity rhythmically. Moreover, UMPS inactivation caused by BMAL1 knockdown plays a vital role in 5-FU resistance *in vivo* and *in vitro*. These findings identify pyrimidine pathway genes as key regulators of 5-FU chronochemotherapy in CRC. We propose that genetic or pharmacological modulation of clock-related proteins may be a promising way to enhance the chemosensitivity of 5-FU.

The antimetabolite 5-FU exerts its antitumor effects by incorporating into DNA and RNA in the same way as uracil. Resistance to 5-FU is associated with the expression of *UMPS*, *TYMS*, *TYMP*, dihydropyrimidine dehydrogenase, and *P53*, as well as microsatellite instability, in CRC (23–27). In recent years, study has shown that upon coincubation with the antimetabolite, uridine competes with 5-FU to be incorporated into RNA, generating 5-FU resistance (28). Moreover, 5-FU combined with irinotecan and oxaliplatin can improve the response rate of advanced CRC by 40–50% (26). However, alternative treatment strategies need to be developed.

The chronotherapeutic effects of 5-FU were reported to be dependent on patient and animal models, and the associated mechanism is unclear in CRC. Our study confirmed that 5-FU had a diurnal effect on CRC, which was demonstrated using subcutaneous tumor formation in nude mice. The volume and weight of subcutaneous tumors in the HALO16 group were significantly smaller than those in the HALO4 group, consistent with reports that 5-FU has better therapeutic effects at HALO14 than at HALO22. TYMS activity was high and BMAL1 expression was low in HALO22 cells (9). Thus, we speculated that BMAL1 was involved in regulating TYMS activity and associated with 5-FU sensitivity in CRC. This finding is supported by the recent studies which reported that BMAL1 overexpression can increase the sensitivity of CRC to oxaliplatin and paclitaxel in tongue squamous cell carcinoma and gemcitabine in pancreatic cancer (29–31). Due to the lack of molecules that can compensate for the function of core circadian genes, BMAL1 is crucial for maintaining the circadian rhythm (21, 32). However, high expression of BMAL1 is associated with drug resistance (33). These phenomena indicate that the complicated mechanisms of circadian regulation influence the effectiveness of cancer therapy. Hence, chronochemotherapies should be designed considering the properties of individual biomolecules to obtain better therapeutic results.

CRISPR library screening is widely used to filter drug targets and study pathogenic mechanisms of disease (34). However, the identified top hits are often difficult to phenocopy in screened models *via* genetic deletion or overexpression of a single gene due to off-target effects and varied transfection efficiencies (19). In this study, we used genome-wide CRISPR screening to identify the mechanism of 5-FU chronotherapy in SW480 cells. We found that the pyrimidine metabolic pathway was involved in 5-FU chronochemotherapy in CRC. We further revealed that knockdown of pyrimidine pathway-related genes conferred 5-FU resistance, whereas their overexpression improved 5-FU sensitivity in CRC, suggesting the possibility of pathway-centered therapy for tumor intervention.

In this study, we discovered a new pathway involved in the therapeutic effects of 5-FU. Intracellular drug levels are determined *via* drug uptake capacity and drug metabolic efficiency (23). The drug 5-FU enters cells *via* the solute carrier family. The mechanism of 5-FU activation involves conversion to fluorouridine monophosphate (FUMP), either directly through OPRTase or indirectly by fluorouridine (FUR) through the sequential action of UPP and UCK (26). Our data indicated that the cellular levels of 5-FU and pyrimidine metabolites, such as uridine and UMP, were regulated by BMAL1. Previous reports have indicated that certain key enzymes related to 5-FU function display circadian rhythms (9, 35). For example, the levels of dihydropyrimidine dehydrogenase, the rate-limiting enzyme for pyrimidine decomposition in monocytes, increased by nearly 40% from 22:00 h to 00:00 h, when the treatment effect of 5-FU is more pronounced (35). Moreover, TYMS was expressed in a circadian manner in subcutaneously transplanted sarcoma (9), which further corroborates our observations in synchronized CRC cells (**Supplementary Figures 3A–C**), indicating fluctuating activity of pyrimidine-metabolizing enzymes. The reduction in pyrimidine metabolites in shBmal1 cells is also possibly due to the low circadian clock state of BMAL1 knockdown cells, resulting in low substrate synthesis and metabolism (36).

Taken together, this study provides a hitherto uncharacterized mechanism of the role of the biological clock in 5-FU chronochemotherapy. In translational studies, pyrimidine metabolic pathway-related genes, such as *UMPS*, should be further developed as a druggable chronomodulated target for preventing resistance to fluorouracil therapy in CRC patients. We investigated only the potential role of the pyrimidine metabolic pathway in the chrono-based chemotherapeutic effects of 5-FU; the roles of other pathways may also be relevant issues for its chronotherapeutics, which await subsequent assessment. We expect that our results will increase researchers’ attention to individual 5-FU precision treatment for CRC.

## Data Availability Statement

The original contributions presented in the study are included in the article/**Supplementary Material**. Further inquiries can be directed to the corresponding authors.

## Ethics Statement

All animal experiments were performed with the approval of the Institutional Animal Care and Use Committee in Fudan University.

## Author Contributions

CL and RQ conceived the project; CL and YN designed the experiments; YN, XF, JXL, XL, YW, and LH conducted the work and contribute to the data collection and data analysis. YN and CL draft and revised the manuscript. YN, RQ, and CL take responsibility for the integrity of the data analysis. All authors contributed to the article and approved the submitted version.

## Funding

This work was supported by the National Natural Science Foundation of China: No. 81870600 (CL); No. 31871189 (RQ); No. 81802901 (YW).

## Conflict of Interest

The authors declare that the research was conducted in the absence of any commercial or financial relationships that could be construed as a potential conflict of interest.

## Publisher’s Note

All claims expressed in this article are solely those of the authors and do not necessarily represent those of their affiliated organizations, or those of the publisher, the editors and the reviewers. Any product that may be evaluated in this article, or claim that may be made by its manufacturer, is not guaranteed or endorsed by the publisher.

## Abbreviations

5-FU, 5-fluorouracil; BMAL1, Brain and muscle aryl hydrocarbon receptor nuclear translocator-like protein-1; CLOCK, Circadian locomotor output cycles kaput; CRC, Colorectal cancer; CRIPSR, Clustered regularly interspaced short palindromic repeats; ENPP3, Ectonucleotide pyrophosphatase/phosphodiesterase 3; HALO, Hours after light onset; KEGG, Kyoto Encyclopedia of Genes and Genomes; LC-MS, Liquid chromatography with mass spectrometry; RNA-seq, RNA sequencing; TYMS, Thymidylate synthase; UCK2, Uridine-cytidine kinase 2; UMPS, Uridine monophosphate synthetase; UPP2, Uridine phosphorylase 2.
