# Supplementary material for: Genome-wide CRISPR Screening Reveals Pyrimidine Metabolic Reprogramming in 5-FU Chronochemotherapy of Colorectal Cancer
Source: Front Oncol. 2022 Jul 12;12:949715. doi: 10.3389/fonc.2022.949715 (PMC9316589; doi:10.3389/fonc.2022.949715)
Supplement: Supplementary file 1 [file DataSheet_1.docx]

**Materials and Methods**

**Flow cytometry**

Cell lines were harvested after 5-FU treatment for 48 h. For cell death analysis, cells were washed with PBS and resuspended in 200 μL of binding buffer, then incubated with 5 μL PI (Yeasen, Shanghai, China) for 5 min, and finally subjected to flow cytometry analysis.

**RNA sequencing and analysis**

Total samples were prepared for RNA sequencing from BMAL1 knockdown and vehicle SW480 cells treated with or without 5-FU (three samples per group) using TRIzol reagent (Invitrogen, MA, USA) according to the manufacturer’s protocol. RNA integrity was assessed on a Bioanalyzer and RNA 6 000 Nano LabChip Kit (Agilent, California, USA), and high-quality samples were used for library preparation. cDNA synthesis and amplification were performed using TruSeq. After normalization and pooling, libraries were sequenced on a Novaseq 6000 (Illumina) using paired-end 150 bp reads. Libraries were sequenced to a depth of >600 million reads per sample. Reads were aligned using the HISAT2 package. StringTie was used to determine the expression levels of mRNAs by calculating FPKM. The significantly expressed mRNAs and genes were selected with log2 (fold change) > 1 or log2 (fold change) < -1 and with statistical significance (p value < 0.05) by the R package edgeR.

**Chromatin immunoprecipitation (ChIP)**

ChIP assays were performed using a Chromatin Immunoprecipitation Kit (Millipore, Darmstadt, Germany) according to the procedures provided by the manufacturer. Chromatin solutions were precipitated using normal human IgG as a negative control and anti-BMAL1 (CST, Darmstadt, Germany) at 4°C for 4 h. The precipitates were analyzed by qPCR, and the primers used for detecting CLOCK-BMAL1 binding to the *UMPS* promoter region are listed in Table S1.

**Luciferase reporter assay**

HCT116 and 293T cells were transfected with the pGL3 basic luciferase reporter vector (Promega, USA) and transcription factor vector using polyethyleneimine (Sigma, Darmstadt, Germany). Cell extracts were harvested 24 h after transfection. Luciferase was measured by the luciferase reporter assay system (Beyotime, Jiangsu, China).

**Western blot analysis**

Cells were lysed in RIPA buffer with phenylmethylsulfonyl fluoride and Protease Inhibitor Cocktail (Sigma, Darmstadt, Germany). The supernatant was normalized by a BCA protein kit. For immunoblotting, protein extracts were denatured by heating at 95°C for 10 min, separated by SDS-PAGE, and transferred to polyvinylidene fluoride membranes. The membranes were blocked with PBST containing 5% skim milk for 1 h at room temperature and then incubated with primary antibodies in the diluent antibody buffers listed in Table S1 of the supplemental materials at 4°C overnight. An ECL enhanced chemiluminescence substrate kit (Bio-Rad) was used for imaging and quantitation after incubation with secondary antibodies by Totallab software.

**Quantitative real-time PCR analysis**

RNA extraction was isolated from cells or tissues with TRIzol (Invitrogen, MA, USA) according to the manufacturer’s protocol. RNA samples were reverse-transcribed using reverse transcription reagents (Toyobo, Osaka, Japan). Quantitative real-time PCR was performed using SYBR Green PCR master mix (Toyobo, Osaka, Japan). The relative expression level was analyzed by the 2−ΔΔCt method and normalized to the expression of GAPDH. For the standard curve method of qPCR, the relative expression level was calculated by standard sample concentrations. The sequences of the primers used for qRT-PCR are listed in Table S1 of the supplemental materials.

**Genomic DNA sequencing**

Genomic DNA extraction (Merck, USA) and PCR amplification were performed according to Zhang’s protocol. In brief, sgRNA sequences were amplified by PCR using the designed primers listed in Table S1 of the supplemental materials. The amplified PCR product was subjected to DNA gel electrophoresis and then extracted and purified for sequencing library construction with the NEB Next UltralI DNA Library Prep Kit for Illumina (NEB #E7645), followed by HiSeq X deep sequencing.

**Liquid chromatography-tandem mass spectrometry (LC-MS)**

The shnc and shBmal1 cells after treatment with or without 5-FU were extracted in 0.5 mL of ice-cold methanol:H_2_O:CH3CL (3:1:1, v/v/v) followed by sonication in an ultrasonic water bath (MRC, Holon, Israel) for 30 min at a frequency of 40 kHz (25°C). Samples were centrifuged at 12,000 × g for 15 min at 25°C, supernatants were filtered through a 0.2 μM membrane, the supernatants were evaporated to dryness under vacuum, and the residues were dissolved in 1 mL of H_2_O/methanol (1:1, v/v).

Samples were centrifuged (10 min at 12,000 g), and 3 μL of the aqueous phase was retained for analysis. Liquid chromatography-tandem mass spectrometry (LC-MS/MS) was performed as described above. A Shimadzu UHPLC system (Kyoto, Japan) equipped an LC-30AD solvent delivery system. The separation of the compounds was carried out on a Waters ACQUITY UPLC HSS T3 1.8 μM operated at 20°C. The mobile phase, which consisted of 0.1% formic acid in water (A) and acetonitrile (B) as the mobile phase, was delivered at a flow rate of 0.5 mL/min under a gradient program. The gradient system was 0–0.5 min, 2% B; 0.5–2.0 min, 2–90% B; 2.0–3.5 min, 90–90% B; 3.5–5.0 min, 90-2% B. The mass spectra were acquired using a TripleTOF™ 6500 system with a Duo Spray source (SCIEX, Foster City, CA, USA) in negative and positive ESI mode. The optimized parameters for negative and positive modes were as follows: the ion spray voltage was set to 5,500 (positive ion mode) and −4,500 V (negative ion mode); the Turbo V spray temperature, 500°C; nebulizer gas (Gas 1), 50 psi; heater gas (Gas 2), 60 psi; collision gas, medium; the curtain gas was kept at 30 psi; and declustering potential, 80 (positive ion mode) and −80 V (negative ion mode). The collision energy was set at 35 (positive ion mode) and −35 V (negative ion mode), and the collision energy spread was 15 V for MS/MS experiments. The data were analyzed by Peak View Software™ 2.2 (SCIEX, Foster City, CA, USA).


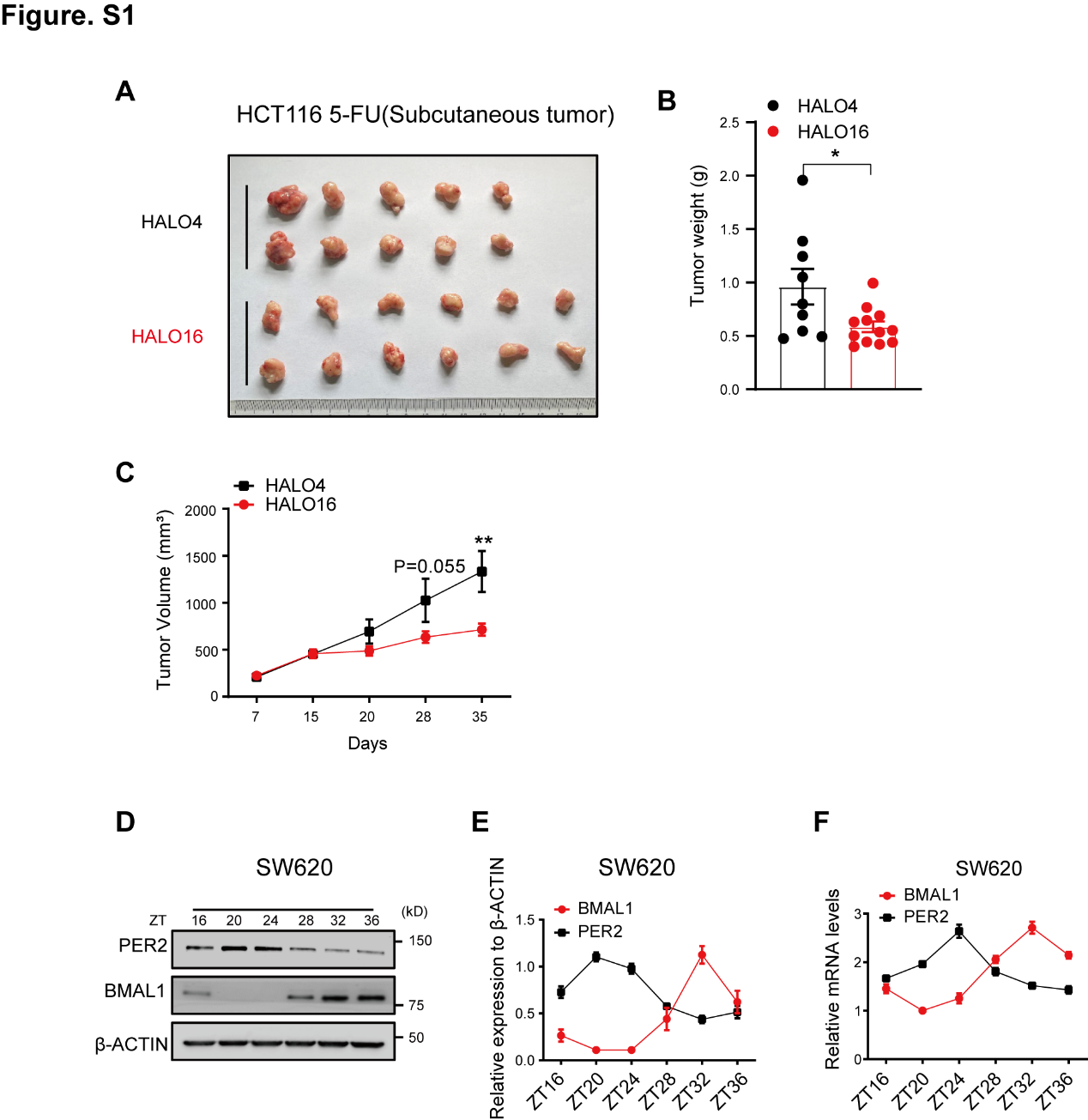


**Figure S1. The therapeutic efficacy of 5-FU in CRC follows the circadian rhythm *in vivo***

**(A, B, C)** HCT116 cells were subcutaneously injected into mice to observe 5-FU chronochemotherapy in cohort 2. One week after cell inoculation, mice were treated with 5-FU (30 mg kg^-1^, twice a week) or PBS at the indicated time points for four weeks. (n = 10 or 12, unpaired t test). * indicates p < 0.05, ** indicates p < 0.01.

**(D, E, F)** The expression of BMAL1 and PER2 at the indicated time points in SW620 cells after synchronization using Western blotting and qPCR (n = 3).


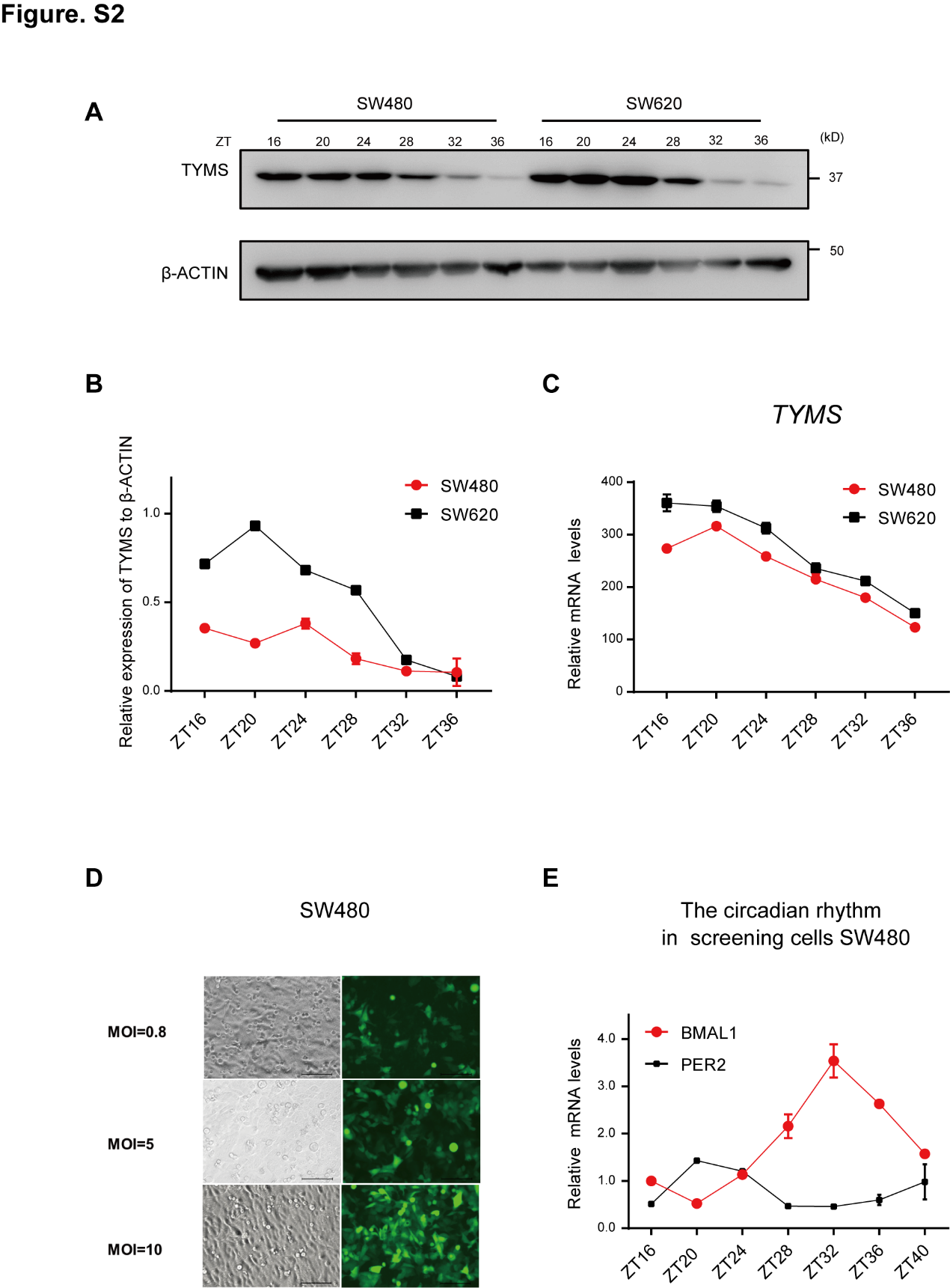


**Figure S2. TYMS, the key enzyme in 5-FU metabolism, is periodic in CRC cells**

**(A, B, C)** The expression of TYMS at the indicated time points in SW480 and SW620 cells after synchronization using Western blotting and qPCR (n = 3). Cells were collected every 4 h for a total of 24 h at the indicated time points. ZT, zeitgeber time (literally, time given or time cue) refers to environmental variables that act as circadian time cues.

**(D)** Premium multiplicity of infection (MOI) searching in lentivirus-transfected SW480 cells (n = 3).

**(E)** Verification of the synchronized efficiency of serum shock in genome-wide CRISPR knockout SW480 cells using qPCR (n = 2). Cells were synchronized with 50% horse serum for 3 h, and 7 samples were collected every 4 h from mice at ZT16.


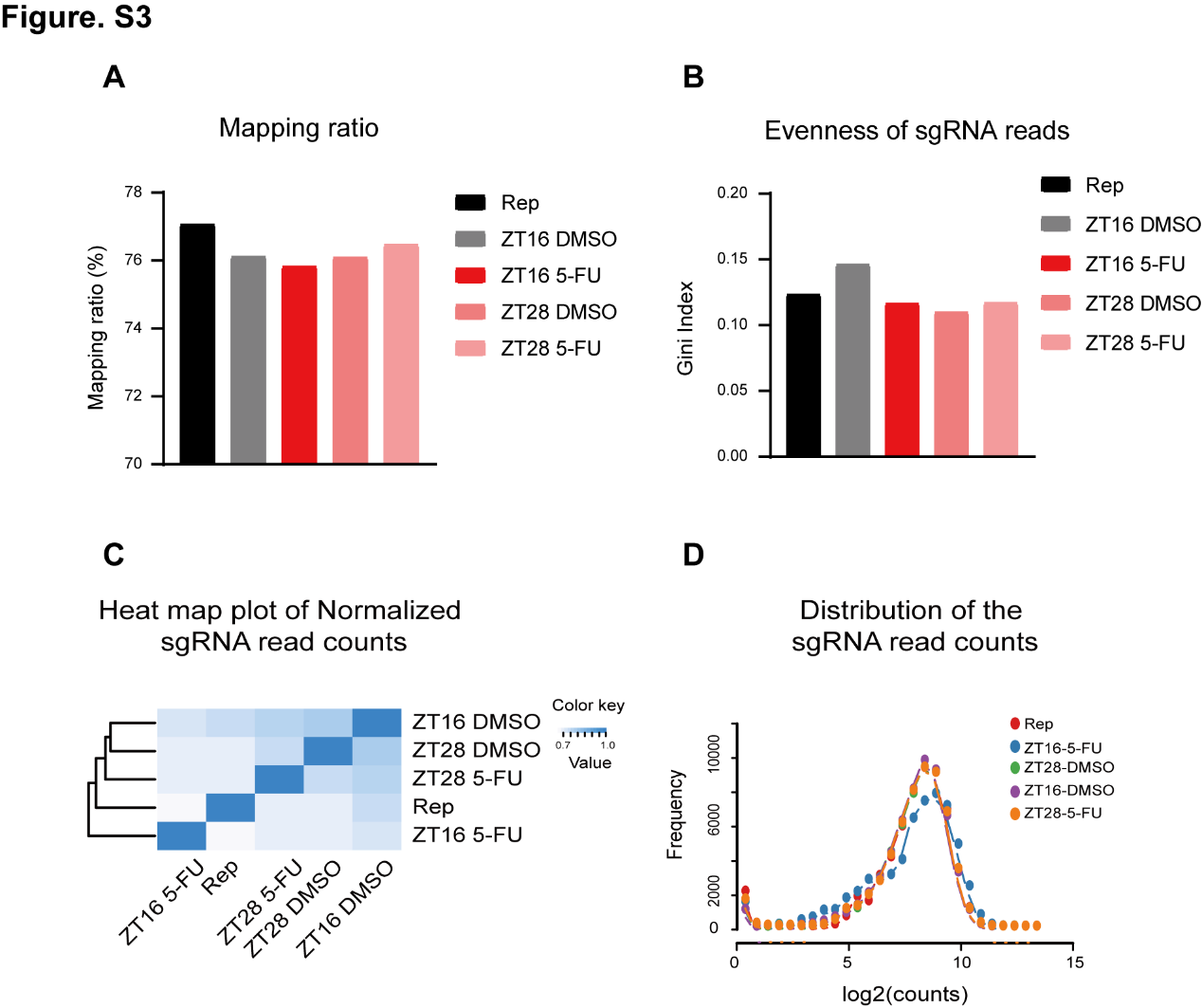


**Figure. S3. Quality control of genome-wide CRISPR screens**

**(A)** Comparison of five groups of mapping ratios. Rep indicates the representative group, namely, cells were collected at ZT0 to represent sgRNA in the genome-wide library. The Rep group indicates baseline cells without any treatment. The ZT16 5-FU group indicates 5-FU treatment at ZT16, and the ZT16 DMSO group indicates DMSO treatment at ZT16. The same was true for the ZT28 5-FU and ZT28 DMSO groups.

**(B)** Comparison of the sequencing uniformity in five groups of sgRNA reads. The Gini index indicates sequencing uniformity, and its value is 0.1-1. The closer the value is to 0.1, the better the uniformity is.

**(C)** The correlation of read counts in the five groups using heatmap.

**(D)** The distribution of sgRNA read counts in five groups.


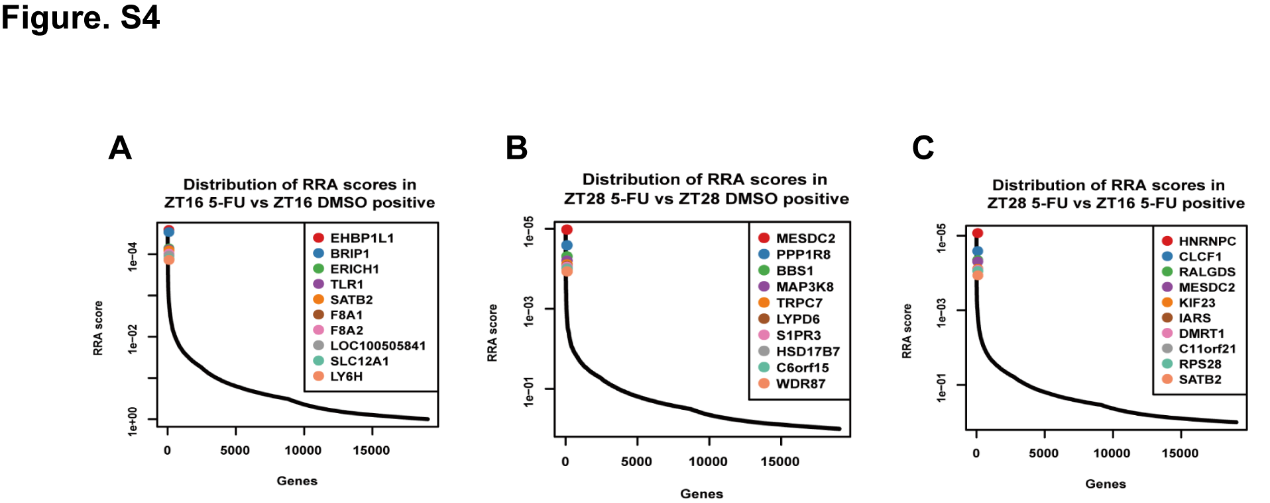


**Figure S4. Analysis of genome-wide CRISPR screening data and preparation of RNA-seq samples**

**(A, B, C)** Top hits in ZT16 5-FU vs. ZT16 DMSO, ZT28 5-FU vs. ZT28 DMSO and ZT28 5-FU vs. ZT16 5-FU analyzed using MAGeCK. Genes: The number of genes in the library; RRA score: Robust rank aggregation score. a method efficiently assessing significant genes.


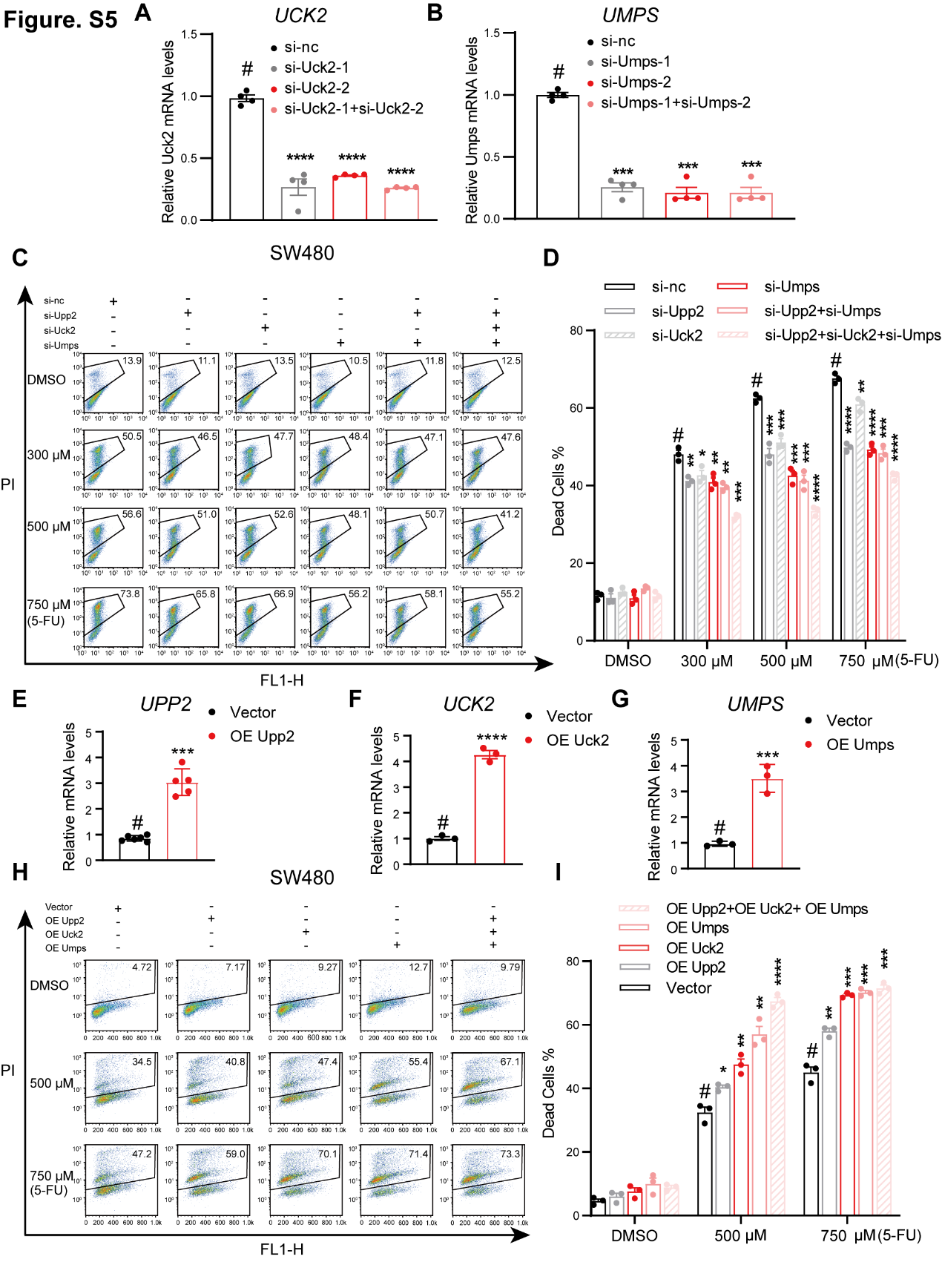


**Figure S5. Genetic deletion of pyrimidine pathway genes promotes robust 5-FU resistance in SW480 cells**

**(A, B)** Validation of the knockdown efficiency of *UCK2* and *UMPS* by qPCR in SW480 cells.

**(C, D)** The number of dead SW480 cells with knockdown of pyrimidine metabolic pathway genes after treatment with 5-FU (0, 300 μM, 500 μM, 750 μM, 48 h) was determined by flow cytometry. (n = 3, one-way ANOVA per concentration).

**(E, F, G)** Validation of the overexpression efficiency of *UPP2*, *UCK2* and *UMPS* by qPCR in SW480 cells.

**(H, I)** Dead cells were determined by flow cytometry of SW480 cells overexpressing pyrimidine metabolic pathway genes after treatment with 5-FU (0, 500 μM, 750 μM, 48 h). (n = 3, one-way ANOVA per concentration). ** indicates p < 0.01, *** indicates p < 0.005, **** indicates p < 0.001 versus si-nc (#) or vector (#), ns indicates that there is no significance.


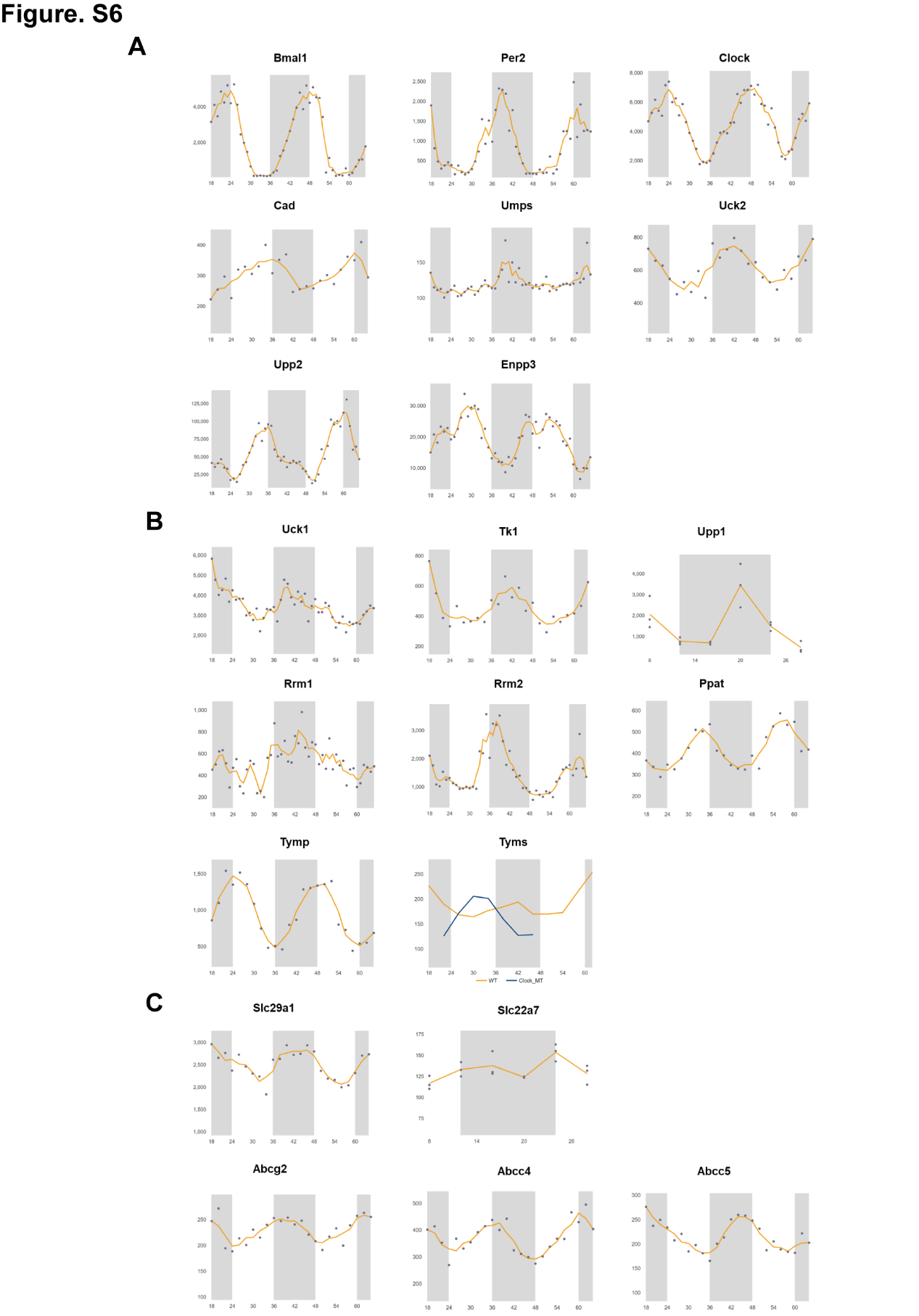
**Figure S6. Diurnal expression of pyrimidine metabolic pathway genes in the Circa database**

**(A)** The circadian rhythm of pyrimidine metabolism pathway genes identified by CRISPR screening (a database of mammalian circadian gene expression profiles, http://circadb.hogeneschlab.org/).

**(B)** The expression pattern of other key metabolic genes in the pyrimidine metabolic pathway using Circadb.

**(C)** The oscillation rhythm of pyrimidine transport carrier genes using Circadb.


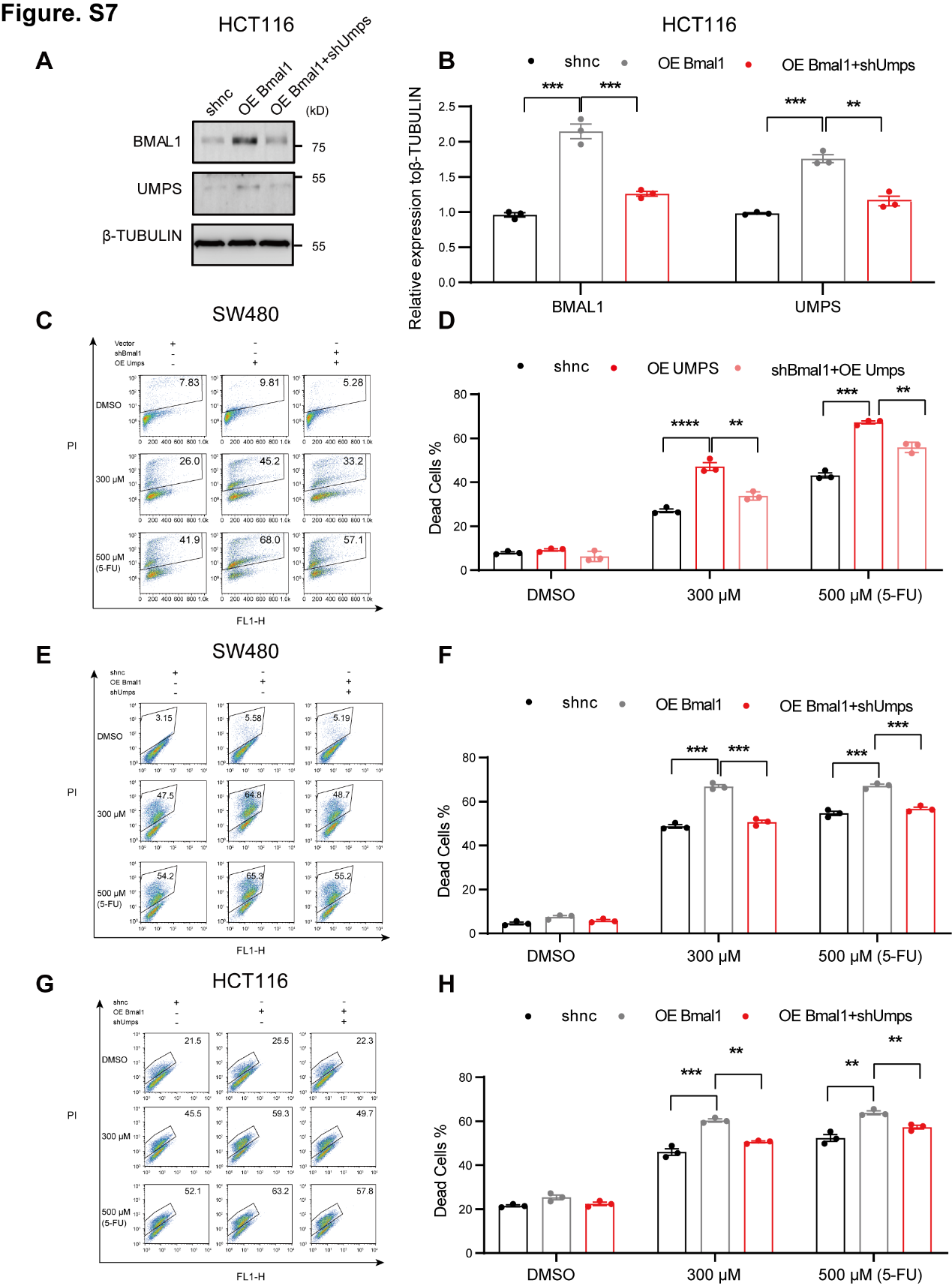


**Figure S7. UMPS enhances 5-FU sensitivity through BMAL1 *in vitro* and *in vivo* in CRC cells**

**(A, B)** The protein expression levels of BMAL1 and UMPS upon simultaneous overexpression of BMAL1 with or without knockdown of UMPS in HCT116 cells via western blotting (n = 3).

**(C, D)** Dead cells were determined by flow cytometry of SW480 cells with simultaneous knockdown of BMAL1 with or without overexpression of UMPS in HCT116 cells after treatment with 5-FU (0, 300 μM, 500 μM, 750 μM, 48 h). (n = 3, one-way ANOVA per concentration).

**(E, F)** Dead cells were determined by flow cytometry of HCT116 cells overexpressing BMAL1 with or without knockdown of UMPS simultaneously after treatment with 5-FU (0, 300 μM, 500 μM, 750 μM, 48 h). (n = 3, one-way ANOVA per concentration).

**(G, H)** Dead cells were determined by flow cytometry of SW480 cells overexpressing BMAL1 with or without knockdown of UMPS simultaneously after treatment with 5-FU (0, 300 μM, 500 μM, 750 μM, 48 h). (n = 3, one-way ANOVA per concentration). ** indicates p < 0.01, *** indicates p < 0.005.

**Supplemental Tables**

**Table S1**

| Gene name | Forward Primer | Reverse Primer |
| --- | --- | --- |
| h-*GAPDH* | GGGAAACTGTGGCGTGAT | GAGTGGGTGTCGCTGTTGA |
| h-*BMAL1* | TGGATGAAGACAACGAACCA | TAGCTGTTGCCCTCTGGTCT |
| h-*PER2* | GACATGAGACCAACGAAAACTGC | AGGCTAAAGGTATCTGGACTCTG |
| h-*CLOCK* | AATGATGACCTTCTTTGCACCA | CAACACCAACCAAGATCCCGA |
| h-library | CGATACAAGGCTGTTAGAGAGA | TCAAGTTGATAACGGACTAGCC |
| m-β-*Actin* | GTGACGTTGACATCCGTAAAGA | GCCGGACTCATCGTACTCC |
| m-*Per2* | GAAAGCTGTCACCACCATAGAA | AACTCGCACTTCCTTTTCAGG |
| m-*Bmal1* | TGACCCTCATGGAAGGTTAGAA | GGACATTGCATTGCATGTTGG |
| h-*ENPP3* | TTTGGGAGGCCTAGGGTACT | TTGGCTCTCAGAAGGAGGAA |
| h-*UPP1* | TGATTGCCCCGTCAGACTTTT | CACCAACGCACCTGATGAAG |
| h-*UPP2* | CCCCAACAGAATGAAAGC | TGACTGATGGCGAGCACA |
| h-*UMPS* | TTGGTGACGGGTCTGTACGA | GAAGACGCGGTCGAGACAC |
| h-*UCK1* | AGTTGCTGGGACAGAACGAG | CTGCCGTCAGGACCTTGTAG |
| h-*UCK2* | CTGAGCCAGGATAGCTTCTACC | CATACACGGGGATCTGGACTG |
| h-*RRM1* | ACTTCGGCTTTAAGACGCTAGA | GCATGAGTAAACCACCTCTCAGA |
| h-*TYMP* | GGTGTGGGTGACAAGGTCAG | GCAGCACTTGCATCTGCTC |
| h-*UMPS* | TTGGTGACGGGTCTGTACGA | GAAGACGCGGTCGAGACAC |
| h-*PER2*-Chip | CTCCCTAGTGATGCGCTTGG | CTCTGAGGTGCCTGTGGTCG |
| h-*UMPS*-Chip | TCATGAAAGGTGGGACTTCA | GCCTCGTCTGCAGCTTTC |
| m-*Tyms* | GGAAGGGTGTTTTGGAGGAGT | GCTGTCCAGAAAATCTCGGGA |

**Table S2**

| Antibodies | Souce | Identifier |
| --- | --- | --- |
| Anti-BMAL1 | CST | 14020 |
| Anti-CLOCK | Abcam | AB3517 |
| Anti-PER2 | Abcam | AB79813 |
| Anti-β-ACTIN | Proteintech | 66009-1-Ig |
| Goat anti-mouse IgG | Proteintech | SA00001-1 |
| Goat anti-rabbit IgG | Proteintech | SA00001-2 |
| Anti-β-TUBLIN | Affinity | T0023 |
| Anti-TYMS | Abcam | AB108995 |
| Anti-UMPS | Novus | NBP2-20810 |
